# Inhalation Dose and Source Term Studies in a Tribal Area of Wayanad, Kerala, India

**DOI:** 10.1155/2017/1930787

**Published:** 2017-05-22

**Authors:** Reshma Bhaskaran, Ravikumar C. Damodaran, Visnuprasad Ashok Kumar, Jojo Panakal John, Danalakshmi Bangaru, Chitra Natarajan, Bala Sundar Sathiamurthy, Jose Mundiyanikal Thomas, Rosaline Mishra

**Affiliations:** ^1^Government Medical College, Kozhikode, Kerala, India; ^2^Department of Physics, University of Calicut, Malappuram, Kerala, India; ^3^Department of Physics, Fatima Matha National College, Kollam, India; ^4^Radiological Safety Division, Indira Gandhi Center for Atomic Research, Kalpakkam, India; ^5^Environmental Assessment Division, Bhabha Atomic Research Center, Mumbai, India

## Abstract

Among radiation exposure pathways to human beings, inhalation dose is the most prominent one. Radon, thoron, and their progeny contribute more than 50 per cent to the annual effective dose due to natural radioactivity. South west coast of India is classified as a High Natural Background Radioactivity Area and large scale data on natural radioactivity and dosimetry are available from these coastal regions including the Neendakara-Chavara belt in the south of Kerala. However, similar studies and reports from the northern part of Kerala are scarce. The present study involves the data collection and analysis of radon, thoron, and progeny concentration in the Wayanad district of Kerala. The radon concentration was found to be within a range of 12–378 Bq/m^3^. The thoron concentration varied from 15 to 621 Bq/m^3^. Progeny concentration of radon and thoron and the diurnal variation of radon were also studied. In order to assess source term, wall and floor exhalation studies have been done for the houses showing elevated concentration of radon and thoron. The average values of radon, thoron, and their progeny are found to be above the Indian average as well as the average values reported from the High Natural Background Radioactivity Areas of Kerala. Exhalation studies of the soil samples collected from the vicinity of the houses show that radon mass exhalation rate varied from below detectable limit (BDL) to a maximum of 80 mBq/kg/h. The thoron surface exhalation rate ranged from BDL to 17470 Bq/m^2^/h.

## 1. Introduction

Among all the natural sources of radiation dose to mankind, inhalation of radon (^222^Rn), thoron (^220^Rn), and their progeny contribute about 50% of global effective dose [1]. Epidemiological studies enhance our understanding of the health risk to ionising radiation. Initially the epidemiological studies from the miners were used to assess the health risk from exposure to radon. However, in the recent past several case control studies of residential exposure to radon have also been reported. These studies also show an increasing risk of lung cancer with increasing exposure to radon [2]. Thoron, on the other hand, has not been studied in detail with reference to lung cancer risk [3]. Only recently the contribution of thoron to the radiation dose has been recognised [4–7]. The south west coast of India is very well studied in terms of both terrestrial radioactivity and the radon and thoron concentrations [8, 9]. However studies in the northern part of Kerala, especially the Malabar region, are scarce. No study of terrestrial radioactivity or indoor radon, thoron, and progeny concentration is so far reported from the Wayanad district of Kerala. Wayanad is one of the coldest high range tourist places situated in the Western Ghats of southern India. Several studies have proved the increase in the concentration of indoor radon with decreasing temperature [10–12]. These studies have reported indoor radon thoron levels which are higher than those seen in the High Natural Background Radioactivity Areas (HNBRA) of Kerala. Hence the present investigations in Wayanad district are imperative to analyse the effect of temperature and ventilation on these gases with respect to the warmer regions of Neendakara and Chavara which are one among the six HNBRAs of the world.

The location we have chosen for the present investigations is Bathery region of Wayanad. This region has large number of granite quarries and granite is already known to possess higher radioactivity when compared to other types of rocks [13–15]. It may be of interest to know the effect of granites in terms of radiation dose, when used as construction material for building. Wayanad is the native place for a large population of tribal community of Kerala who belong to low income group. These people live in houses made of construction materials like mud available locally. The study aims to analyse the effect of the locally available construction materials comprising granites and mud on the inhalation dose due to radon and thoron to the residents of Bathery Taluk.

## 2. Materials and Methods

### 2.1. Geology

Wayanad district is situated on the southern tip of the Deccan plateau and forms a part of the Western Ghats. The geographical coordinates for the district are 11°36′18′′N and 76°04′59′′E. The population is 816558 as per the 2011 census and the area is 2132 km^2^. The altitude ranges from 700 to 2100 meters above sea level. The mean annual temperature is 23.8°C. During December–January temperature lowers to 15° C. Bathery is located at 11.67°N 76.28°E. It has an average elevation of 907 m. Ambalavayal is part of Bathery and is one of the main regions having a large number of granite quarries. The Ambalavayal granite is of four types, namely, foliated granite, pink granite, grey granite, and aplitic granite [16]. These are composed of quartz, pink feldspar, hornblende, and biotite.

### 2.2. Materials

Indoor radon and thoron measurements were divided into two phases of one year each. In the first phase (2014-2015), pinhole dosimeters were used for the measurement of indoor radon and thoron concentrations. This study included all the villages in Bathery. From this study spatial variation of radon and thoron in Bathery was assessed. Along with the indoor radon and thoron concentration studies, soil samples were collected from the top surface (of 15 cm thickness) from the vicinity of the houses. These samples were analysed using gamma ray spectrometry for assessing terrestrial radioactivity. Radon and thoron exhalation studies were also done on the samples to obtain the mass exhalation rate for radon and the surface exhalation rate for thoron. In the second phase, 19 houses in the Ambalavayal region were studied during 2015-2016. Out of these 10 houses were intensively studied using both active and passive measurement techniques. The second-phase study involved the measurement of both radon and thoron concentrations as well as their progeny. The diurnal variation of radon and wall and floor exhalation was also studied by using active measurement techniques.

#### 2.2.1. Passive Measurement

LR115 films based pinhole dosimeters [17] were used for the passive measurement of radon and thoron concentration. This device has a single face for gas entry and gives the time integrated measurement of radon and thoron. Pinhole dosimeters equipped with LR-115 Type II Solid State Nuclear Track Detectors (SSNTD) with 2 × 2 cm^2^ area were hung in a room of the houses (at least 30 cm away from nearby wall) for a period of three months. The dosimeters were hung at a height of around 2 m from the floor. The principle, working, and processing of the dosimeter are discussed in detail elsewhere [17, 18]. The dosimeters were replaced with new ones at the end of every three months until a cycle of 1 year was completed. The retrieved dosimeters were processed in the laboratory and the developed tracks were scanned using a spark counter by the standardised protocol. The minimum detectable limit for radon in pinhole dosimeter is 2 Bq/m^3^ and that for thoron is 6 Bq/m^3^.

Progeny measurements were done using the deposition based Direct Radon Progeny Sensors and Direct Thoron Progeny Sensors (DRPS/DTPS) [19]. DTPS is made of LR-115 SSNTD mounted with absorbers in 50 *μ*m aluminised Mylar. This detects the alpha particle emitted from ^212^Po (thoron progeny with energy 8.78 MeV). The radon progeny sensor DRPS is made of LR115 mounted with the absorber thickness 37 *μ*m. This detects 7.69 MeV alpha particles emitted from ^214^Po. The minimum detectable limit for radon progeny in DRPS is 1 Bq/m^3^ and that for thoron progeny in DTPS is 0.1 Bq/m^3^. The principle and method of measurement of radon and thoron progeny is discussed in detail by Prasad et al. [20].

#### 2.2.2. Active Measurements

The active measurements were done using the state-of-the-art equipment, the Portable Radon Monitor (SMART RnDuo), developed by BARC (Bhaba Atomic Research Center, Mumbai). The detector works on the principle of scintillation counter by scintillation with ZnS(Ag). The detection limit of Portable Radon Monitor (SMART RnDuo) is in between 8 Bq/m^3^ and 50 MBq/m^3^ (Operational Manual of Portable Radon Monitor—SMART RnDuo, March 2015). The detector has a progeny filter that eliminates the radon and thoron progenies. For the estimation of radon concentration, thoron is eliminated by the diffusion time delay based thoron discriminator which takes advantage of the short half-life of thoron.

For thoron monitoring, program based sampling is carried out using a flow mode sampler connected to the pump inlet of the monitor. Each measurement cycle consists of 15 minutes. During this the pump is kept ON for the first 5 minutes. This gives a measure of the thoron and the background. A delay of 5 minutes is maintained after this to ensure the complete decay of thoron. The last 5 minutes gives a measure of the background count for the cycle. The background count is subtracted from the total count of the initial 5 minutes to get the thoron concentration.

#### 2.2.3. Gamma Ray Spectrometry and Exhalation Studies

Soil samples were collected from the top 15 cm of the surface soil. These samples were then dried, sieved, and sealed in a hermetically tight container and kept for 30 days to attain secular equilibrium between the radon and its parent nuclide. NaI (Tl) scintillation detector available at the Radiological Safety Division of Indira Gandhi Center for Atomic Research (IGCAR) was used for gamma ray spectrometry of the sources. The method for estimation of the terrestrial radioactivity using NaI (Tl) detectors is discussed in detail in many papers [21–23].

The radon and thoron exhalation studies were done by using the enclosed sample method as explained by Petropoulos et al. [24]. RAD 7 detector [25, 26] manufactured by Durridge Inc. USA was used for the soil exhalation studies. It detects the radon and thoron concentrations, between 4 and 400,000 Bq/m^3^. The methodology suggested by the manufacturer was followed. The mass exhalation rate of radon was calculated using the formula [22, 27–29].

(1) $$\begin{array}{c} J_{M}=C_{0}\cdot e^{-\lambda \cdot t}+\frac{C\cdot V}{M\cdot t}\cdot \frac{\lambda \cdot t}{1-e^{-\lambda \cdot t}}, \end{array}$$

where *C*_0_ is the initial radon concentration (Bq/m^3^), *C* is the accumulated radon concentration, and *J*_*M*_ is the mass exhalation rate (mBq/kg/h). Accumulated time is denoted by *t* (h), *V* is the effective volume in m^3^ (volume of the container + volume of the detector − volume of the sample), *M* is mass of the sample in kg, and *λ* denotes the effective decay constant of radon, which is the sum of the leak rate (if existing) and the radioactive decay constant of radon (h^−1^).

The thoron measurement is also done by keeping the sample in a closed chamber. The thoron surface exhalation rate is calculated using the formula [29].

(2) $$\begin{array}{c} J_{s}=\frac{C_{T}\cdot V\cdot \lambda }{A}. \end{array}$$

Here *C*_*T*_ is the equilibrium thoron concentration which is attained in a short time period. *V* is the residual air volume of the setup in m^3^, *λ* is the thoron decay constant (0.0126 s^−1^), and *A* is the surface area (m^2^) of the sample.

The soil gas radon activity measurements were done to study the contribution of the radon gas in the subsoil to the indoor radon concentration. The soil gas measurement was done at a depth of 0.8 m as it is already known from the literature that the activity concentration of radon in the soil increases with increasing depth up to around 1 m from the surface and then it reaches saturation [30, 31]. The soil gas radon activity measurements were done in ten houses (selected based on indoor radon and thoron concentration, availability of power point, and safety of the equipment) where the active measurements were conducted using Portable Radon Monitor SMART RnDuo. A steel probe was inserted into the soil for measurement. The measurement cycle was set at 15 minutes. Soil gas was sampled for 2 minutes using a pump before starting the measurement. The measurement is continued for 1 hour to have a stabilised reading of radon concentration.

The health effect due to radon and thoron exposure was quantified in terms of the annual effective dose *E*. Annual effective dose *E* was calculated as [32].

(3) $$\begin{array}{c} E=C\times Q\times F\times t, \end{array}$$

where *C* (in Bq m^−3^) is the concentration of isotope of interest, *Q* (nSv per Bq h m^−3^) the dose conversion factor (9 nSv per Bqhm^−3^ for radon and 40 nSv per Bqhm^−3^ for thoron), and *F* the equilibrium factor (EF) for radon and thoron (*F*_*R*_ and *F*_*T*_, resp.). *t* is the total time of exposure calculated by using an indoor occupancy factor of 0.8 (*t* = 7000 hr/y). *F* is calculated as follows [20]:

(4) $$\begin{array}{c} F=\frac{EEC}{C}. \end{array}$$

Here, EEC is the equilibrium equivalent concentration of radon and thoron (EERC and EETC, resp.) calculated as given by Prasad et al. [20]. *C* is the concentration of radon or thoron as the case may be.

## 3. Results

**Figure 1:**
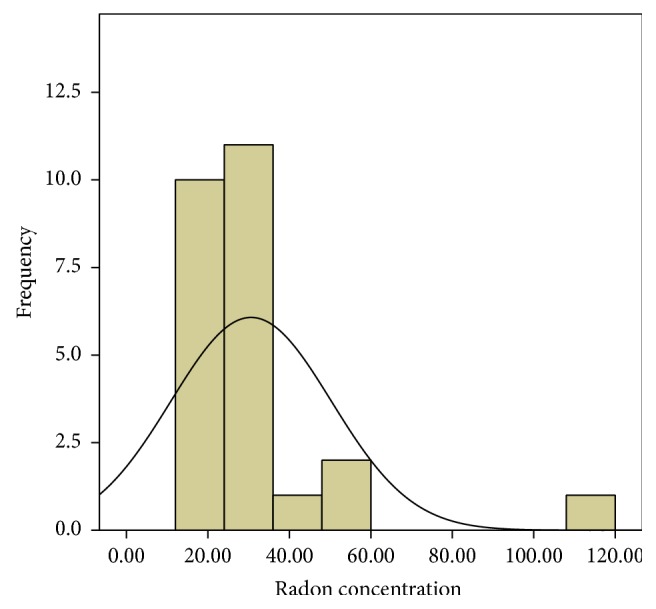
Distribution of radon concentration in phase 1 study.

**Figure 2:**
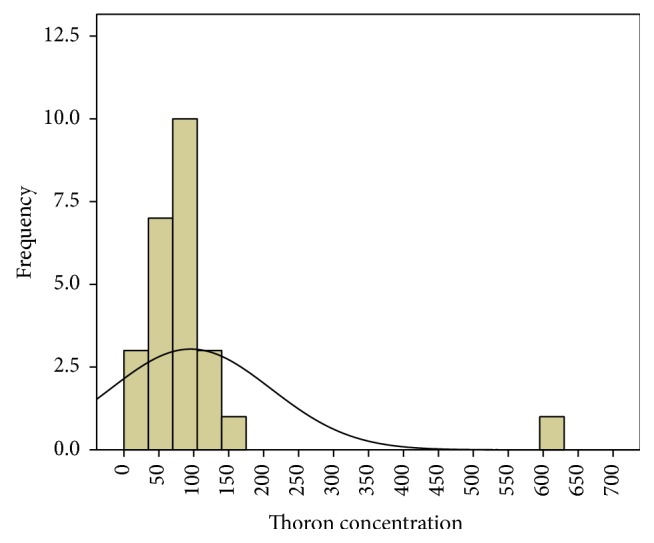
Distribution of thoron concentration in phase 1 study.

**Figure 3:**
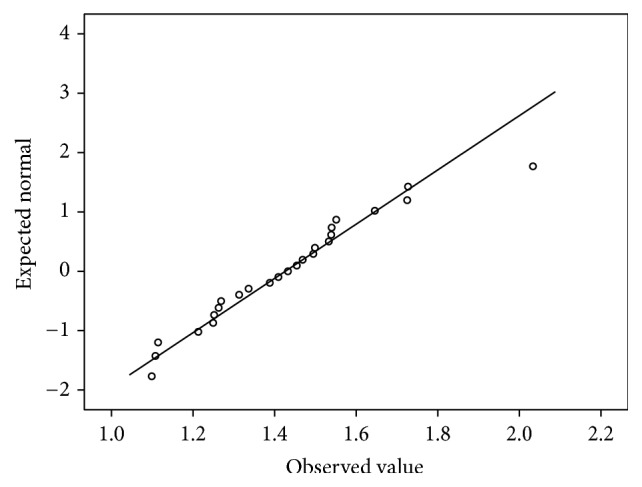
Q-Q plot for radon concentration.

**Figure 4:**
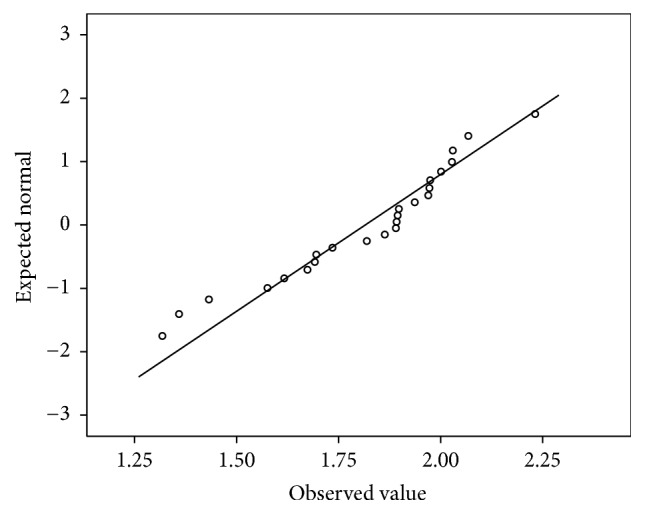
Q-Q plot for thoron concentration.

In the first phase, 25 houses were studied in Bathery. The average of radon and thoron concentration for a year is as given in Table 1. The average ratio of thoron to radon is 3.3. It is seen that the thoron concentration in one house is highly elevated (annual average thoron concentration = 621 Bq/m^3^) as compared to all the other houses. As this house is very close to the granite quarries, intensive study was planned in the houses in the vicinity of this house and those near other granite quarries. The frequency distribution of both radon and thoron has a positive skewness as shown in Figures 1 and 2. The Q-Q plot of the logarithm of radon concentration for the 25 samples is shown in Figure 3. The same is repeated for thoron excluding the outlier and given in Figure 4. The linearity of the plots (Figures 3 and 4) confirms the lognormal distribution of radon and thoron concentrations. A similar observation of lognormal distribution of radon and thoron concentration was made in the HNBRA of Kerala and some other studies [8, 33].

### 3.1. Radon, Thoron, and Progeny Measurements

To find the concentration of radon and thoron progeny, in the second phase, measurements were carried out in the vicinity of the house showing high concentration of thoron as well as a few houses in the vicinity of other granite quarries. The average of the radon, thoron, and progeny measurements for the second phase is as given in Table 2. The average equilibrium factor determined for the region is 0.7 for radon and 0.04 for thoron. The EF is higher for radon as compared to the UNSCEAR value of 0.4. The equilibrium factor for thoron is also greater than the value (0.02) given in UNSCEAR [34]. Similar result of higher EF for radon and thoron as compared to the world average or the HNBRA of south west coast of India is found in the Beijing area of China. In the Chinese study, it is seen that the EF increases during the period of Haze-fog [35].

**Figure 5:**
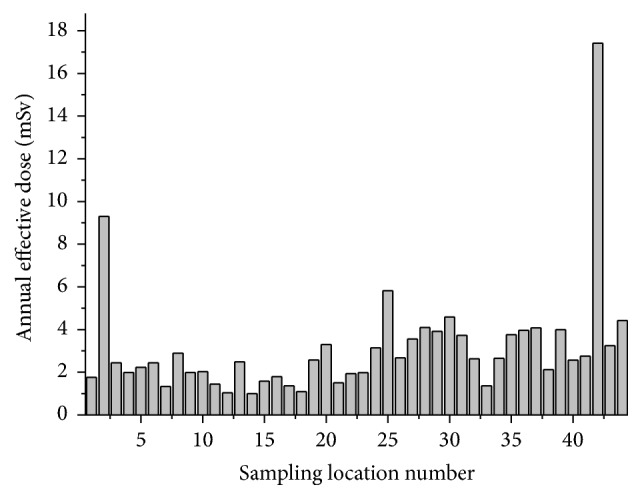
Inhalation dose to the residents of Bathery, Wayanad, Kerala.

The inhalation dose for the residents of the region using the average equilibrium factor for radon and thoron is as given in Figure 5. From the study of the radon and thoron concentration it is seen that, out of the 44 houses studied, only one house [2.3%] showed radon concentration greater than the action limit prescribed by International Commission on Radiological Protection, ICRP (300 Bq/m^3^).

### 3.2. Active Measurements

**Figure 6:**
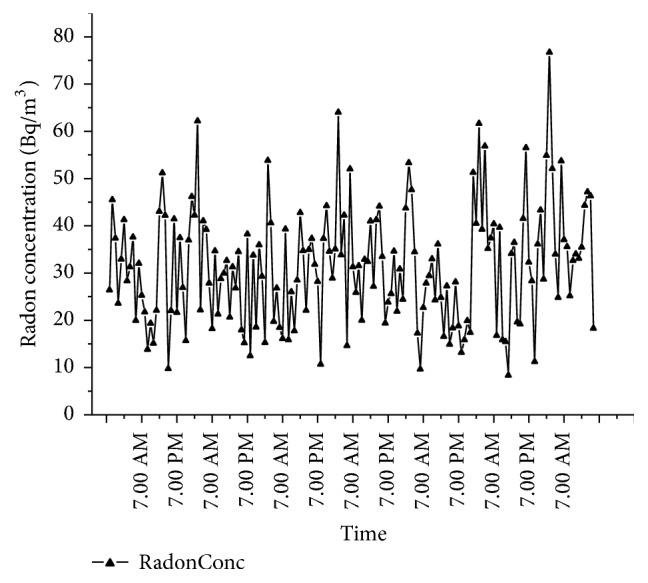
Diurnal variation of radon in one house for 5 days.

**Figure 7:**
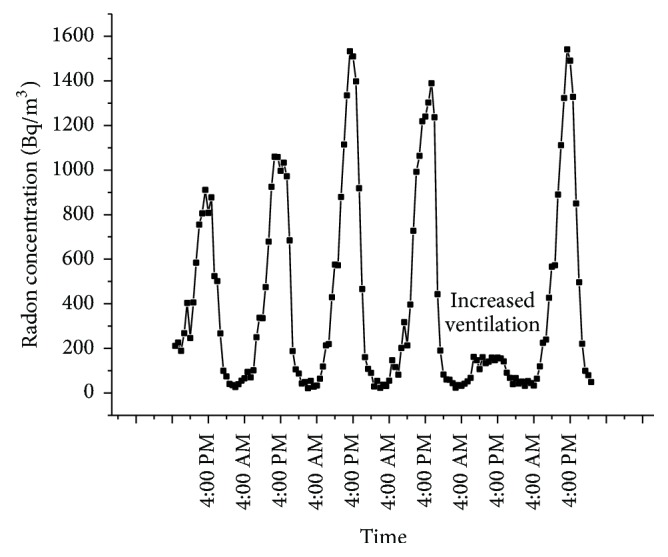
Diurnal variation of radon in the house showing maximum radon concentration.

As the passive measurements using the pinhole dosimeters may be influenced by factors like the bulk etching rate, leakage from the cup, and so forth [36], active measurements were done to ascertain and validate the concentration obtained using pinhole dosimeters. Ten houses including the houses showing higher radon and thoron concentrations were intensively studied. The active measurements were done for a period of five days in every house to study the diurnal variation. Plot of the diurnal variation is as given in Figure 6. It is seen that the concentration of radon reduces during the day time when the houses are more ventilated as compared to the night. There is no particular pattern and the concentration keeps fluctuating during day time. The maximum concentration of radon is found after midnight and in the early morning hours. In the house showing the highest concentration of radon, the value increased to as high as 1533 Bq/m^3^ at night (Figure 7). However, the concentration dropped to levels shown during the day time when the room had increased ventilation by keeping the door of the room open overnight.

Figure 7 shows the significant variation which ventilation can make to the concentration of radon. The maximum concentration of radon decreased by a factor of 5 when the door was kept open along with the window opposite to it in the corridor which opens to the outside environment.

### 3.3. Exhalation Measurements

As part of phase 1 study, soil samples were collected from the vicinity of the houses. As mentioned earlier, since majority of the houses in this area are made up of mud blocks procured from the site of construction itself, the aim of collecting the soil samples was to study the correlation, if any, between indoor radon thoron concentration and the radon thoron exhalation rate from the soil samples. From the gamma ray spectrometry of 25 soil samples, it is observed that the ^238^U concentration varies from below detectable limit (BDL) to 68 Bq/kg with an average of 18 ± 15 Bq/kg. The ^232^Th concentration ranges from BDL to 112 Bq/kg with an average of 40 ± 29 Bq/kg.

**Figure 8:**
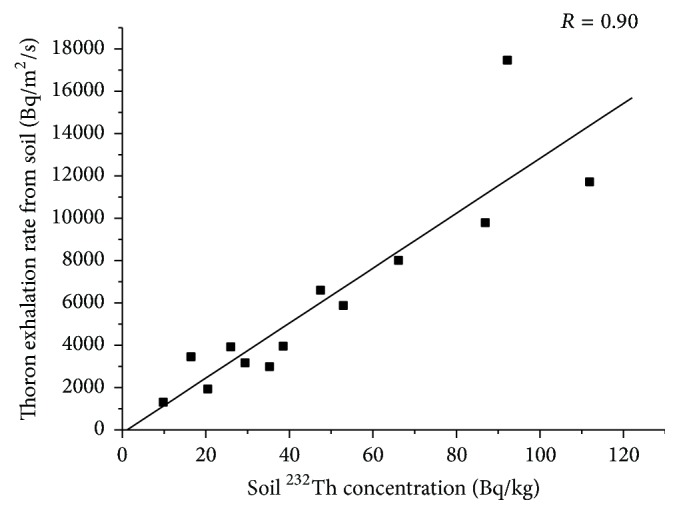
^232^Th versus thoron exhalation rate in soil.

Exhalation studies using RAD 7 were done on the 25 samples. Out of the 25 samples, 14 had radon mass exhalation rate as BDL. 12 samples also showed BDL values for thoron surface exhalation rate. The radon mass exhalation rate varied from BDL to a maximum of 80 mBq/kg/h. The thoron surface exhalation rate ranged from BDL to 17470 Bq/m^2^/h. No correlation was found between the indoor radon concentration and radon mass exhalation rate from soil. Similarly, no correlation was found between indoor thoron concentration and the thoron surface exhalation rate. No correlation was found between radon exhalation rate from soil and ^238^U concentration in the soil. However, as seen in Figure 8, there is a good correlation between thoron exhalation rate and ^232^Th concentration in soil.

To analyse the source term for the relatively higher concentration of radon and thoron, exhalation studies were done for the walls and floor in the houses showing the highest concentration of radon and thoron. The result of the exhalation studies for radon shows that the exhalation rate is more from the floor (4.39 ± 0.19 Bq/m^2^/hr) as compared to the walls (1.34 ± 0.12 Bq/m^2^/hr). The house showing high concentration of thoron was also studied by wall and floor exhalation. It is found that the walls are the major source of thoron as compared to the floor (mean wall and floor contribution being 5.81 ± 0.20 and 2.53 ± 0.18 Bq/m^2^/s, resp.).

Soil gas radon activity measurements were done to analyse the contribution of the soil gas under the houses to the indoor radon concentration. A maximum of 28 kBq/m^3^ radon concentration in the soil gas was found in the vicinity of the house showing maximum indoor radon concentration. Only marginal variation (22 kBq/m^3^ to 28 kBq/m^3^) was found in the radon concentration in the soil gas at the various sites. This value falls in the category of soil with normal radon potential as per the Swedish criteria [37].

**Figure 9:**
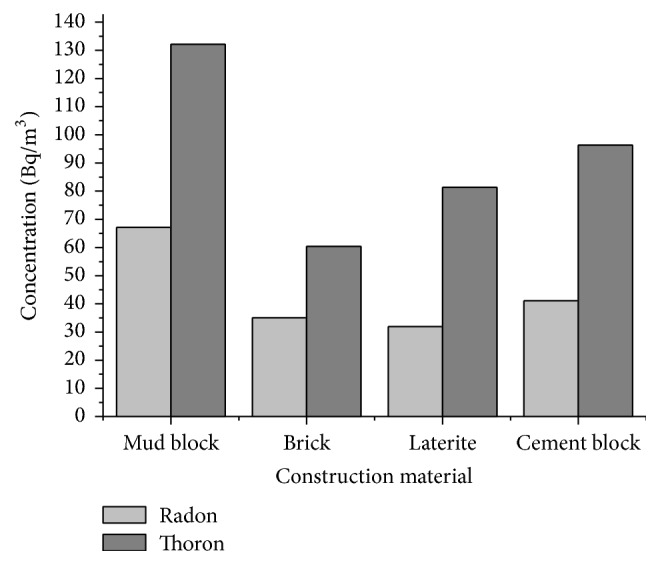
Variation of indoor radon and thoron concentration with construction material.

A study of the indoor concentration of radon and thoron with respect to the construction material shows that the mud houses have the highest concentration of radon (Figure 9). Both the houses which showed highest indoor radon and thoron concentration are mud houses with very poor ventilation. Ventilation estimation was done by taking into consideration the number of doors and windows in the room where the dosimeters were kept. Rooms with only one door and no window were called poorly ventilated. Most of such houses had the door of the room facing another room or corridor. Thus the exchange of air from the outside environment was comparatively less. Rooms with one door and one window, which were kept open for a major part of a day, were considered to be moderately ventilated. Rooms with more than one door and more than one window which were kept open for a large fraction of a day were assumed to be well ventilated.

## 4. Discussion

### 4.1. Radon, Thoron, and Progeny Measurements

The initial study of the indoor radon and thoron concentration shows that the thoron concentration in the region is higher compared to the radon concentration. This may be due to the fact that majority of houses in this region are made up of building materials which are locally available. The region is one of the major suppliers of the granite and mechanised sand made out of crushed granite to Malabar as well as the neighbouring districts of Tamil Nadu and Karnataka states. It is well known that the concentration of ^232^Th is higher in granite due to the presence of monazite [14]. Hence the higher concentration of thoron may be attributed to the presence of higher concentration of thorium in the building materials. The study of indoor radon and thoron concentration in the Neendakara-Chavara region, which is one of the HNBRAs of the world, shows a median of 23 Bq/m^3^ for radon and 24 Bq/m^3^ for thoron [38]. Even though Neendakara-Chavara region has very high terrestrial radioactivity due to the presence of monazite sand, the indoor radon thoron levels are less as compared to the present study. This result is in agreement with the discussion by Mishra and Sapra in the BARC newsletter [9]. Here they argue that even though the HNBRA of Kerala experiences 10 times more external gamma effective doses, the inhalation doses due to indoor radon and thoron concentrations are similar to the Normal Background Radiation Areas (NBRA). The reasons provided by them are the excessive ventilation and the type of flooring which shields the radon from reaching the indoor environment in the HNBRA. Our study proves that along with this the construction material of the houses in the HNBRA may also be contributing to the reduction in the radon thoron concentrations as compared to the present study. Unlike Wayanad, the construction material for the coastal region of Neendakara-Chavara, mainly consisting of sand, may be coming from outside the region which may not be part of HNBRA. Hence the radioactivity content in these construction materials may not be as high as the sand in the HNBRA. Even in the present study the average value of radon and thoron concentration increases when the region of interest is concentrated near the granite quarries. The workers in the quarries mainly reside in this area. Houses are of old style made of mud blocks and with very less ventilation. The temperature in the southern part of Kerala is higher (average of 30°C to 35°C) as compared to the Wayanad district which is one of the coldest tourist places in South India. Due to the colder climate people here opt for lesser ventilation as compared to the houses in the warmer regions of Kerala. Hence lack of ventilation may also be one of the reasons for the increase in indoor radon thoron concentration as compared to the HNBRA of Kerala. Similar results are available from the Tehri-Garhwal regions of India which also are high altitude regions and hence colder places. The radon concentration in these places is reported to be an average of 100 Bq/m^3^ in winter and that of thoron is 126.4 Bq/m^3^ [18]. As seen in Table 2, the average EF for radon is more as compared to the value given in UNSCEAR [23]. The EERC values are effected by both natural and mechanical ventilation [39]. The houses in the present study were selected based on their type of construction. Majority of the houses had poor ventilation for energy efficiency. The life style in the area does not involve artificial ventilation or air conditioners. Besides, as the study was done near the areas of the granite quarries, the dust loading may also contribute to the accumulation of progeny in the houses. This may be the reason for the higher EERC and hence higher EF of radon. The thoron EF is also more as compared to the value mentioned in UNSCEAR [34]. The concentration of thoron may be less at the centre of the rooms as compared to the walls due to its very short half-life [40]. As the pinhole dosimeters were hung in the center of the room, the concentration of thoron would have been less and nonuniform over the period of three months.

The study shows that only one house has radon concentration greater than the limit prescribed by ICRP [41]. As no such limit is prescribed for thoron so far, the concept of equivalent radon concentration for thoron is used to estimate the hazard due to thoron as suggested by Chen and Moir [42]. The equivalent radon concentration, *R*_Tn_, is the radon concentration that delivers the same annual effective dose as resulted from the thoron concentration *C*_Tn_:

(5) $$\begin{array}{c} R_{\text{Tn}}=\frac{C_{\text{Tn}}\times F_{\text{Tn}}\times 7000\times 40}{F_{\text{Rn}}\times 7000\times 9}. \end{array}$$

**Figure 10:**
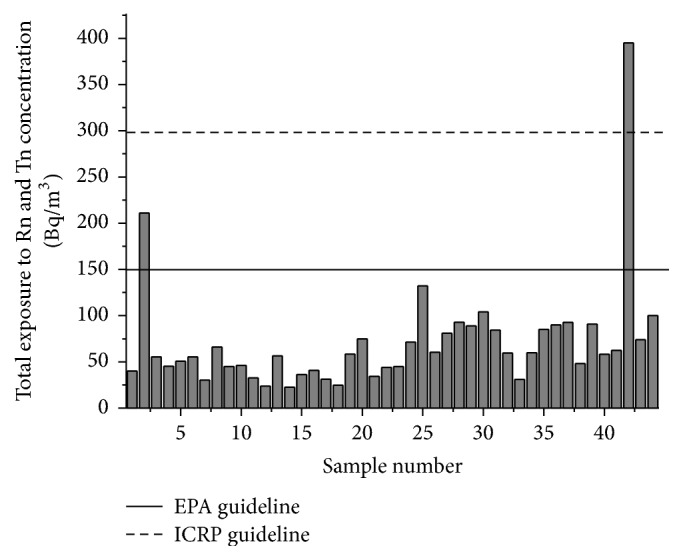
Distribution of exposure to total radon concentration (radon concentration + equivalent radon concentration for thoron concentration).

The total indoor exposure to radon and thoron may be regarded as the sum of the radon concentration and the equivalent radon concentration for thoron. This total exposure can then be compared to the radon guideline value. The equivalent radon concentration as given by Jing Chen and Deborah Moir was calculated for all the samples. Out of the 44 samples only two samples showed values for total exposure to radon and thoron greater than the limits prescribed by Environmental Protection Agency, US (150 Bq/m^3^). Only one sample showed values greater than the limits for indoor radon concentration prescribed by ICRP (Figure 10).

### 4.2. Active Measurements and Exhalation Studies

From the gamma ray spectrometry of soil samples from vicinity of houses it was observed that the average thorium concentration is more as compared to the average uranium concentration. However, no significant correlation was found between the indoor radon concentration and the exhalation rate of radon from soil. Similarly, no significant correlation was found between the exhalation rate of thoron and the indoor thoron concentration. The reason for this may be the variation in the ventilation condition of the houses. Since the concentration of radon varies significantly with the ventilation conditions, even in houses with high exhalation rate of radon, the concentration of indoor radon may not be very high or may be even low if well ventilated.

Besides this, variation in the type of building and flooring material may also affect the indoor radon concentration and hence the correlation may be lost. In the case of thoron along with the factors mentioned for radon the short half-life may also contribute to the lack of correlation. From Figure 8 we see that very good correlation was found between ^232^Th and thoron surface exhalation rate whereas no such correlation was found between ^238^U and radon mass exhalation rate. Since radon is the daughter product of the ^238^U series, a correlation between ^238^U and radon exhalation rate is expected. However, no significant correlation was observed, most likely due to the small sample size.

The diurnal variation shows that the concentration of radon builds up at night when the ventilation in the houses is significantly reduced. The maximum concentration was found in the early mornings. In the house showing highest concentration of radon, the concentration increased during the early morning hours. The concentration starts dropping once the residents wake up and open the door to the room (which happens to be the only ventilation in the room). As the average radon concentration is higher than the limits prescribed by International Commission for Radiation Protection (ICRP) [41], the residents of the house were advised to increase the ventilation in the room by affixing an exhaust fan. Another room in the house was also studied using active measurements to check whether the concentration is the same in all the rooms. However, this room showed lesser concentration (average 161 Bq/m^3^). The room showing the highest concentration of radon had very large cracks in the floor. The exhalation studies of the walls and floor showed that the major contribution to the radon concentration came from the floors. Due to the closed room, at night the temperature inside the room will be higher as compared to the outside environment. This results in stack effect, drawing the radon inside the room. The large cracks on the floor further assists in the flow of radon from soil gas to the indoor atmosphere due to the pressure variation between the indoor areas and the soil gas. Besides this the room has only one door giving entry to another room and not the outside environment. Hence lack of air circulation from the outdoor environment may also be contributing to accumulation of radon at night when the door was kept closed. As the second room has intact floors and the door of the room faces the outside environment, the concentration of radon was not as high as seen in the first room. This shows that in spite of the source (concentration of radon in the soil gas) being the same, cracks and openings in the floor and walls contribute significantly to the increase of the radon concentration and hence the inhalation dose to the residents. A similar effect was seen in the case of thoron. The exhalation study of the walls and floors of the house showing higher thoron concentration showed that the contribution from the walls was more as compared to the contribution from the floor. In this case, also the cracks in the walls may be one of the reasons for the excessive exhalation of thoron. In this study, nearly all the houses had tiled roof or roof made of asbestos. Hence the contribution from roof was not taken into consideration.

Comparison of the radon and thoron concentration with respect to the construction material used showed that the maximum concentration of radon and thoron was found in the houses made of mud blocks. In most of the houses, mud required for the mud blocks was taken from the site of construction itself. Wayanad district has a major population of tribal community who are below poverty line and reside in houses made of mud. The architecture involves very less ventilation to conserve energy. Hence a detailed study of the radon and thoron in the tribal villages may lead to better understanding of the inhalation dose to the residents due to radon and thoron. The modern houses are all well ventilated and hence the radon thoron levels are within the permissible limits.

## 5. Conclusion

The study of radon, thoron, and progeny concentration of Bathery region of Wayanad district shows that the average concentration of radon and thoron is greater than the values observed in the HNBRA. The average equilibrium factor for radon as well as thoron is greater than the values of world average reported in UNSCEAR. No correlation was found between indoor radon concentration and radon mass exhalation rate of soil. The correlation between indoor thoron concentration and thoron surface exhalation rate of soil was also found to be feeble. The concentration of radon seems to increase with decreasing ventilation. Among the various types of building materials mud houses show higher concentration of both radon and thoron. These results suggest that the soil in the vicinity of the houses has very little contribution to the indoor radon thoron concentration as compared to the type of building material and existing ventilation conditions. Radon and thoron concentration was found to be higher in houses close to granite quarries in comparison with those farther away or in urban area. 2.5 per cent of the houses showed radon concentration greater than the reference limit prescribed by ICRP. The study points towards the need for analysis of a larger sample to give a better picture of the radon thoron concentration in the whole of Wayanad district. The Kalpetta region, which is also famous for the Kalpetta granites, should also be studied in detail to understand the variation these granites make, as compared to the Ambalavayal granites, to the indoor radon thoron concentration, when used as construction material.

## Figures and Tables

**Table 1:** Average concentration of radon and thoron in Wayanad, Kerala, India. Result of phase 1 study.

	Radon [Bq/m^3^]	Thoron [Bq/m^3^]
Minimum	13	21
Maximum	108	621
Average	31	96
Standard deviation	20	115
Geometric mean	27	72
Geometric standard deviation	2	2

**Table 2:** Average concentration of radon, thoron, and progeny in Wayanad, Kerala, India. Result of phase 2 study.

	Maximum	Minimum	Average ± SD	GM (GSD)
Radon concentration [Bq/m^3^]	379	20	68 ± 74	54 (2)
Thoron concentration [Bq/m^3^]	576	16	123± 115	95 (2)
EERC [Bq/m^3^]	160	10	43 ± 30	37 (2)
EETC [Bq/m^3^]	8	1	3 ± 2	3 (2)
*F*_Rn_	0.89	0.43	0.69 ± 0.14	0.68 (1.24)
*F*_Tn_	0.14	0.01	0.04 ± 0.03	0.03 (1.83)

## References

[1] UNSCEAR (2000). Sources and effects of ionising radiation.united nations scientific committee on effect of atomic radiation. *Report to the General Assembly*.

[2] SENES Consultants Limited for Canadian Nuclear Safety Commission, “Estimation of the Range of Radiation Dose for a Radon Progeny Working Level Due to Physical Parameters,” 2013

[3] Wiegand J., Feige S. (2002). Thoron: ignored and underestimated in the big shadow of radon—an example from China. *Geofisica Internacional*.

[4] Shang B., Chen B., Gao Y., Wang Y., Cui H., Li Z. (2005). Thoron levels in traditional Chinese residential dwellings. *Radiation and Environmental Biophysics*.

[5] Ramola R. C., Gusain G. S., Rautela B. S. (2012). Levels of thoron and progeny in high background radiation area of southeastern coast of Odisha, India. *Radiation Protection Dosimetry*.

[6] Saïdou, Tokonami S., Janik M., Samuel B. G., Abdourahimi, Joseph Emmanuel N. N. (2015). Radon-thoron discriminative measurements in the high natural radiation areas of southwestern Cameroon. *Journal of Environmental Radioactivity*.

[7] Tokonami S. (2010). Why is 220RN (thoron) measurement important?. *Radiation Protection Dosimetry*.

[8] Chougaonkar M. P., Eappen K. P., Ramachandran T. V. (2004). Profiles of doses to the population living in the high background radiation areas in Kerala, India. *Journal of Environmental Radioactivity*.

[9] Mishra R., Sapra B. K. (2015). Apprehensions about inhalation exposures due to radon (222 Rn) and thoron (220 Rn) in high background radiation areas of india. *BARC Newsletter*.

[10] Kandari M. S., Ramola R. C. (2009). Analysis of seasonal variation of indoor radon concentration in Tehri Garhwal, Northern India. *Indian Journal of Physics*.

[11] Prasad M., Rawat M., Dangwal A., Prasad G., Mishra R., Ramola R. C. (2016). Study of radiation exposure due to radon, thoron and progeny in the indoor environment of yamuna and tons valleys of garhwal himalaya. *Radiation Protection Dosimetry*.

[12] Ramola R. C., Choubey V. M., Prasad Y., Prasad G., Bartarya S. K. (2006). Variation in radon concentration and terrestrial gamma radiation dose rates in relation to the lithology in southern part of Kumaon Himalaya, India. *Radiation Measurements*.

[13] Ningappa C., Hamsa K. S., Reddy K. U., Niranjan R. S., Rangaswamy D. R., Sannappa J. (2016). Study on radon concentration at the work places of mysuru, bengaluru and kolar districts of karnataka state, South India. *Radiation Protection Dosimetry*.

[14] Sannappa J., Ningappa C., Narasimha K. N. P. (2010). Natural radioactivity levels in granite regions of Karnataka State. *Indian Journal of Pure and Applied Physics*.

[15] Ningappa C., Sannappa J., Karunakara N. (2008). Study on radionuclides in granite quarries of Bangalore rural district, Karnataka, India. *Radiation Protection Dosimetry*.

[16] Anilkumar P. S., Varma A. D. K., Nair M. M. (1993). Detailed studies on acid intrusives of Kerala, Part-B. *GSI Report*.

[17] Sahoo B. K., Sapra B. K., Kanse S. D., Gaware J. J., Mayya Y. S. (2013). A new pin-hole discriminated 222Rn/220Rn passive measurement device with single entry face. *Radiation Measurements*.

[18] Joshi V., Dutt S., Yadav M., Mishra R., Ramola R. C. (2016). Measurement of radon, thoron and their progeny concentrations in the dwellings of pauri garhwal, Uttarakhand, India. *Radiation Protection Dosimetry*.

[19] Mishra R., Rout R., Prajith R., Jalalluddin S., Sapra B. K., Mayya Y. S. (2016). Innovative easy-to-use passive technique For 222 Rn And 220 Rn decay product detection. *Radiation Protection Dosimetry*.

[20] Prasad M., Rawat M., Dangwal A. (2016). Variability of radon and thoron equilibrium factors in indoor environment of Garhwal Himalaya. *Journal of Environmental Radioactivity*.

[21] Bangotra P., Mehra R., Kaur K., Jakhu R. (2016). Study of natural radioactivity (226Ra, 232Th and 40K) in soil samples for the assessment of average effective dose and radiation hazards. *Radiation Protection Dosimetry*.

[22] Bala Sundar S., Chitra N., Vijayalakshmi I. (2015). Soil radioactivity measurements and estimation of radon/thoron exhalation rate in soil samples from Kalpakkam residential complex. *Radiation Protection Dosimetry*.

[23] Harb S., Abbady A. E.-B., El-Kamel A. E.-H., Saleh I. I., Abd El-Mageed A. I. (2012). Natural radioactivity and their radiological effects for different types of rocks from Egypt. *Radiation Physics and Chemistry*.

[24] Petropoulos N. P., Anagnostakis M. J., Simopoulos S. E. (2001). Building materials radon exhalation rate: ERRICCA intercomparison exercise results. *Science of the Total Environment*.

[25] Ahmad N., Jaafar M. S., Khan S. A., Nasir T., Ahmad S., Rahim M. (2013). Measurement of radon exhalation rate, radium activity and annual effective dose from bricks and cement samples collected from Dera Ismail Khan. *American Journal of Applied Sciences*.

[26] Pant P., Kandari T., Prasad M., Ramola R. C. (2016). A comparative study of diurnal variation of radon and thoron concentrations in indoor environment. *Radiation Protection Dosimetry*.

[27] Hassan N. M., Hosoda M., Iwaoka K. (2011). Simultaneous measurement of radon and thoron released from building materials used in Japan. *Progress in Nuclear Science and Technology*.

[28] Mann N., Kumar A., Kumar S., Chauhan R. P. (2016). Measurement of indoor radon-thoron in air and exhalation from soil in the environment of Western Haryana, India. *Radiation Protection Dosimetry*.

[29] Singh L. M., Kumar M., Sahoo B. K., Sapra B. K., Kumar R. (2016). Study of radon, thoron exhalation and natural radioactivity in coal and fly ash samples of kota super thermal power plant, Rajasthan, India. *Radiation Protection Dosimetry*.

[30] Antonopoulos-Domis M., Xanthos S., Clouvas A., Alifrangis D. (2009). Experimental and theoretical study of radon distribution in soil. *Health Physics*.

[31] Hafez Y. I., Awad E.-S. (2016). Finite element modeling of radon distribution in natural soils of different geophysical regions. *Cogent Physics*.

[32] UNSCEAR (2000). Annex B. Exposures from natural radiation sources, united nations scientific committee on effect of atomic radiation. *Report to the General Assembly*.

[33] Yamada Y., Sun Q., Tokonami S. (2006). Radon-thoron discriminative measurements in Gansu province, China, and their implication for dose estimates. *Journal of Toxicology and Environmental Health - Part A*.

[34] UNSCEAR 2006

[35] Hou C., Shang B., Zhang Q., Cui H., Wu Y., Deng J. (2015). Impact of haze-fog days to radon progeny equilibrium factor and discussion of related factors. *Radiation and Environmental Biophysics*.

[36] Eappen K. P., Mayya Y. S. (2009). Factors affecting the registration and counting of alpha tracks in solid state nuclear track detectors. *Indian Journal of Physics*.

[37] WHO, “Man-made Mineral Fibres and Radon, IARC monograph Volume 43,” 1983

[38] Pereira C. E., Vaidyan V. K., Chougaonkar M. P., Mayya Y. S., Sahoo B. K., Jojo P. J. (2012). Indoor radon and thoron levels in Neendakara and chavara regions of southern coastal Kerala, India. *Radiation Protection Dosimetry*.

[39] Singh M., Singh K., Singh S., Papp Z. (2008). Variation of indoor radon progeny concentration and its role in dose assessment. *Journal of Environmental Radioactivity*.

[40] Ramachandran T. V. (2010). Environmental thoron (^220^Rn): A review. *Iranian Journal of Radiation Research*.

[41] (2007). International Commission on Radiation Protection. *ICRP Publication 103, Ann. ICRP*.

[42] Chen J., Moir D. (2012). The concept of equivalent radon concentration for practical consideration of indoor exposure to thoron. *International Journal of Environmental Research and Public Health*.

